# 

**Published:** 1880-12

**Authors:** 


### CONTENTS FOR DECEM'R, 1880.

ORIGINAL COMMUNICATIONS.

ART. I.—Surgeons of Baltimore. By B. BERNARD BROWNE, M. D. .... 559
ART. II.—Gunning on Jaw Fractures. By PROF. JAMES E. GARRETTSON, M. D. .... 562
ART. III.—Treatment of Fracture of the Jaw, with Critical Remarks. By THOMAS BRIAN GUNNING, D. D. S. .... 564
ART. IV.—Poisoned by Vegetables from Poisoned Soils. By W. R. MUNROE, M. D. .. 575

CORRESPONDENCE.

Advertising in Medical College Announcements and in Newspapers .... 576

EDITORIAL DEPARTMENT.

Teaching and Licensing .... 578
Sewage of Baltimore, and the National Board of Health .... 579
Close of Volume First .... 580
Syrup of Hypophosphites .... 581
To Correspondents .... 582
A Full Index List of Contributors .... 582

BIBLIOGRAPHICAL DEPARTMENT.

The Art of Prolonging Life .... 582
Explanation of a Simple Method for the Diagnosis for Organic Valvular Diseases of the Heart 583
The Journal of Nervous and Mental Diseases.... 583
A Practical Treatise on Mechanical Dentistry... 584
HYMENEAL .... 585
NATALITIAL, NECROLOGICAL .... 586

EXCERPTA.

A New Anti-Neuralgic: Menthol .... 587
Subcutaneous Injection of Ether in Sciatica .... 588
Iodine in Typhoid Fever .... 588
Remedies for Headache .... 589
Creosote in Phthisis .... 591
Blood Stains .... 592
How the Metric System has been Getting on... 592
Antiseptic Treatment of Typhoid Fever .... 595
Professional Aphorisms .... 596
Antiseptic Surgery .... 597
Gratitude of Patients .... 598
The Diphtheria Discussion at the Medical Society 598
Pleuritis Effusion—Treatment by Hypodermic Injections of Pilocarpin .... 599
Simple Method of Reducing Paraphimosis .... 600
Compression of the Feet of Chinese Women .... 600
The Physician in the School-Room .... 601
Atropia against Cardio-Inhibitory Effects of Chloroform .... 602
Treatment of Pulmonary Phthisis by Benzoate of Sodium .... 602
Anæsthetics in Labor .... 604
Is Shampooing Advisable? .... 605
The Mortality of Physicians .... 605
MORTUARY STATISTICS .... 606

